# Supplementary material for: Genome Analysis of Epsilon CrAss-like Phages
Source: Viruses. 2024 Mar 27;16(4):513. doi: 10.3390/v16040513 (PMC11054128; doi:10.3390/v16040513)
Supplement: Supplementary file 1 [file viruses-16-00513-s001.zip › viruses-2908373-Supplementary_Figures.pdf]

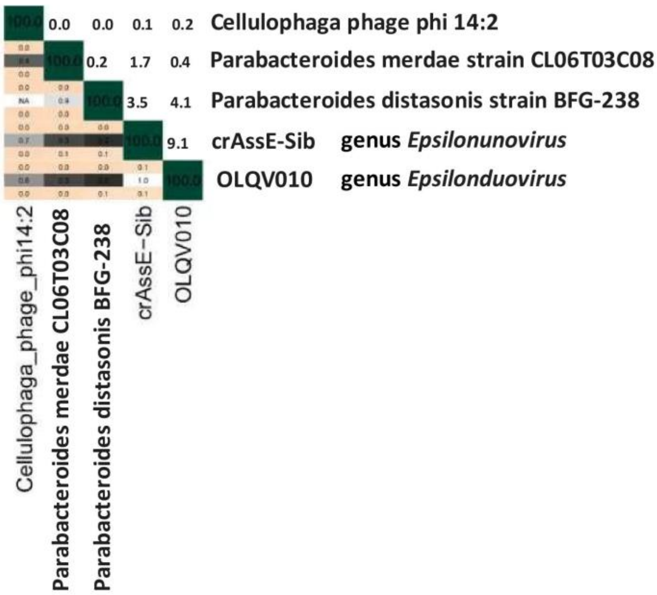

Figure S1. Bidirectional clustering heatmap visualizing VIRIDIC-generated similarity matrix for the crAssE-Sib phage, phage phi14:2, and two prophages.

```
TR1    MRLVEQHIIKQSSIYYNELQDLLHKCKNLYNKGLYVVRQHYFQYKNDNTVKYKYLNYYSLERVLKTENDVDYRAL
TR2    MISYKY--NIYHSKKNKRLIKMLRECCFVWNHALALQRRYYRLFG-----KYIPLNKMQKHF----AKRIKRSIL
       *   :       *   :.* .:*::*  ::*:.* : *::*  :      **  **  . .      : :.   *

TR1    PAPVAQQVLMMVDRNFKSFFNLFNKKNRGEYSEFVRMPKYLNKDGLFPAVFTTASFSQKWIKQGIVKLPKQFSFT
TR2    HSQTAQEILRRLDNAYNRFFKKLSK----------RPPKFKRADKFSSFVFTQSGFTLNGNCFTINKGKKRFKFS
       : .**::*  :*. :: **: :.*          * **: . * : . *** :.*: :     * *  *:*.*:

TR1    -TRTNKQNIQQLRFVPKNGY---IVLEIVYNKKEKDLMSDNGNYLGIDIGLDNLASCVSNNGSCFIINGRPLKSI
TR2    YSRKYEGRIKQVRIVRETCNRFSLIIVTDHNPINNCRKTYDGASTGLDFGLKTYLTKSDGTKIESTLFFKQHQNK
        :*. : .*:*:*:* :.     :::   :*  ::   : :*   *:*:**..  :  ...     :  :  :.

TR1    NQYYN----KRLAFLKS---KLKDNKHTSKQIRSLTNKRNNKIKDYLHKASRILVNHVVSNGINTIIIGHNKCWK
TR2    IRKLHKKLSRAEVGSNNRKRILFDLQQEYRKIRNLRNDFQWKLAHSLCKEY----DYIFIEDLN--IEGMKRLWG
        :  :    :  .  :.    * * ::  ::**.* *. : *: . * *      :::. :.:*  * * :: *

TR1    QEIN-IGKRNNQNFVSIPFNVFISMISYKATLEGINVKIVEESYTSKCSFLDNERICKHESYKGRRTKRGLFKTS
TR2    KKISDLGHSSFINK-----LIFISL-KYDTVVHKID-KWYPSSKTCECGCINKNLSLK---------DRVWICPK
       ::*. :*: .  *       :***: .*.:.:. *: *   .* *.:*. ::::   *         .*  : ..

TR1    FGRTINADINGAFNIIRKSEKESF-DVTMLPEGRGFWWN--PVRISV
TR2    CGAINDRDVLAARNILRKGISELESEGNTSSRNTGVPCVSIQESDSL
       *   : *: .* **:**. .*   : .  ... *.         *:
```

Figure S2. Alignment of the crAssE-Sib transposases sequences.

```
AR1             MGTNVSNFQNNAKNSNIILTSESNEMEFSKEVKTVSSFKNSDFGELKIII
AR2             M-------------------------------DKVSVFESSDFGELRIIV
                *                               ..** *:.******:**:

AR1             ID-EEPYFIGSPIASFLGYTNPRKAIRDHVDEDDRLIMKVPDTQ-GWNET
AR2             DPKGDVWFVASDVAKSLGYINAKDAVKRHVDDDDSILLQVSDNQWGVNQS
                    : :*:.* :*. *** *.:.*:: ***:** ::::*.*.* * *::

AR1             FRPYTPNTKILIINESGLYSLIFGSKMDFAKKFKKWVTSEVLPSIRKTGS
AR2             ILKTRYIDNIRIINESGLYSLILSSKLESAKRFKRWITSEVLPSIRKTGE
                :       :* ***********:.**:: **:**:*:************.

AR1             YSITPKDY----------PSALRALADEIDAKNRAIAERAQAEAERQQAI
AR2             YKTSSGGKGILVPDFSNPADAARAWADQYEAAQKAIAEKSQAEAEKQQAL
                *. :. .           ..* ** **: :* ::****::*****:***:

AR1             KTIEEQRPDVEFAESFKKVDHENMWLIRDVAKKLEQNGIIIAEKNLRLFL
AR2             KTIEEHKPDVEFAESFRKVDHNNMWLIRDIAKKLEQNGVIIAEKNLRSFL
                *****::*********:****:*******:********:******** **

AR1             EEVKFMFRNGQGRWELYSDIVKNKFGVYRSYFVDKYSGERVNQQTIYMTG
AR2             EEAKFMFRNGLGKWELYSNVVVKGYGVYRSYFIDKYSGDRINQQTIYMTG
                **.******* *:*****::* : :*******:*****:*:*********

AR1             AGYEVTLNGIKGKCRNTFLNYGKFEGHNF
AR2             SGYEVTLNGIKGKLKNVFLKYGKFA----
                :************ :*.**:****
```

Figure S3. Alignment of prophage antirepressor sequences.

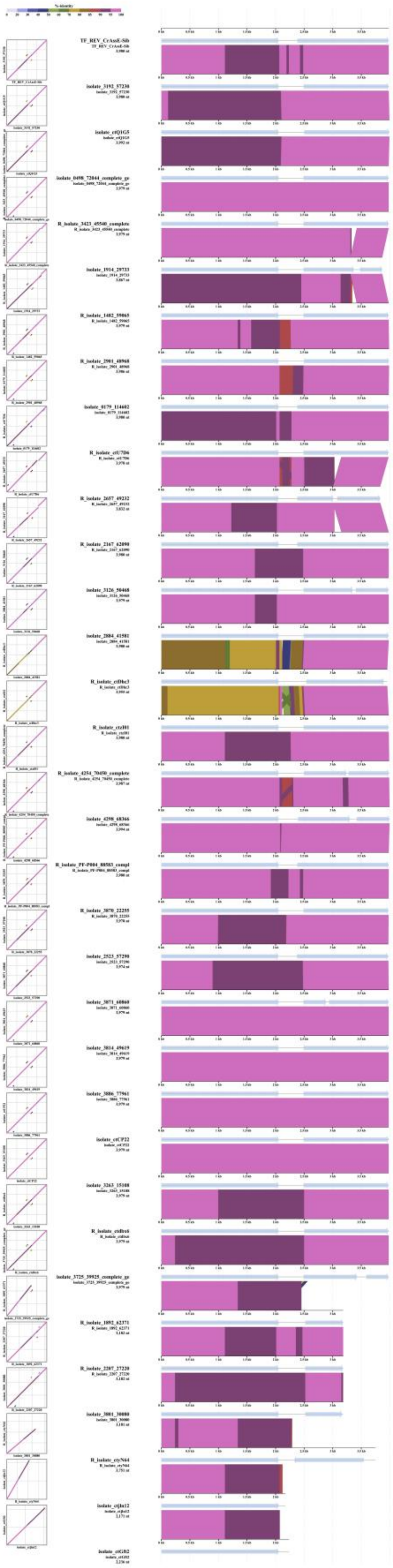

Figure S4. Comparative alignment of the crAssE-Sib genome fragment, including genes receptor binding protein and reverse transcriptase and close phage genome fragments. The percentage of sequence similarity is indicated in color; the color scale is shown at the top. The dot-plot analysis is shown on the left. Analysis was performed using VipTree software.

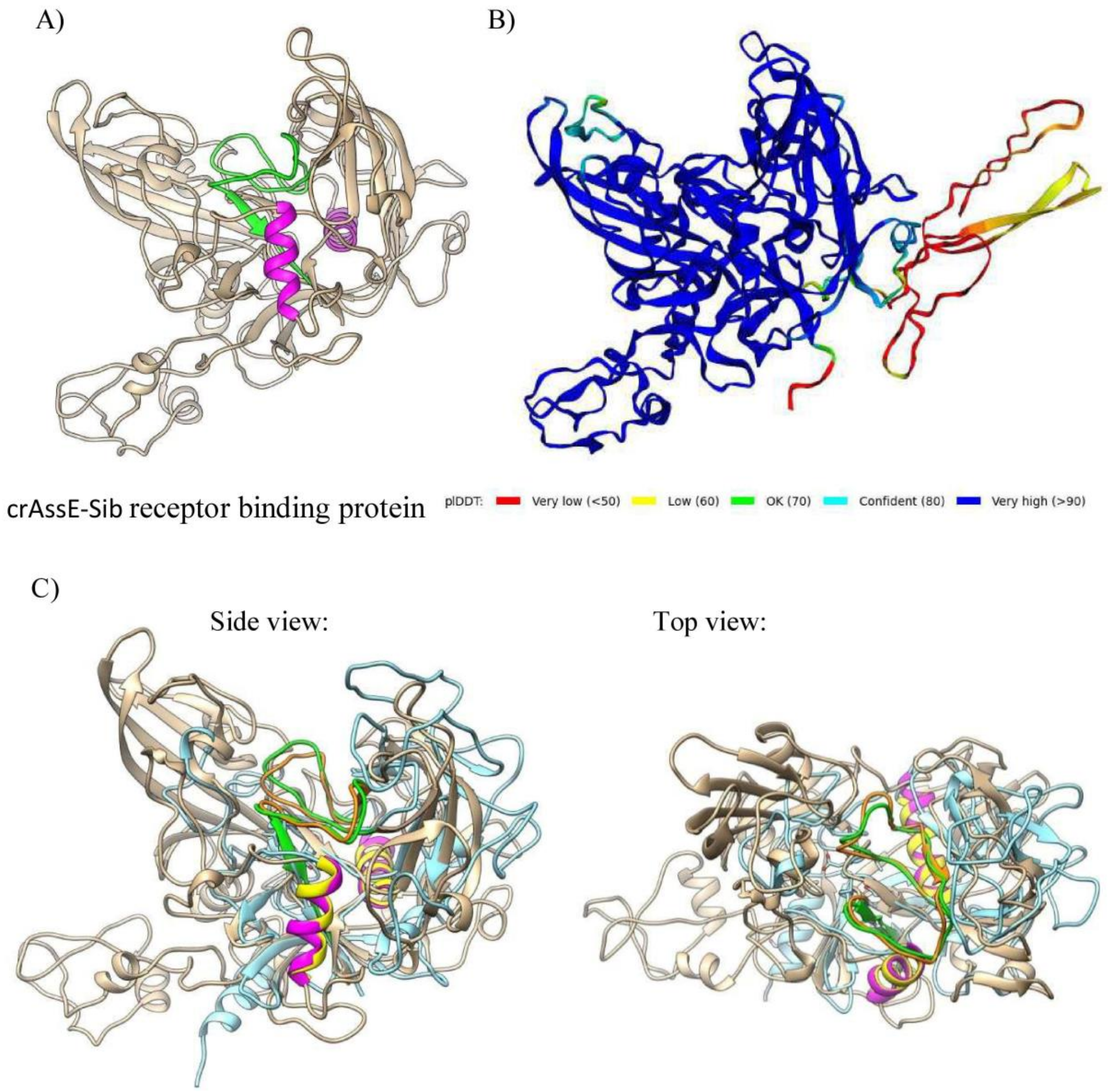

Figure S5. 3D model of crAssE-Sib receptor binding protein (RBD). A) 3D model of crAssE-Sib RBD from 85 aa to 678 aa; VR region is colored in green. B) Alphafold2 model of this protein with probability that is shown by color according to pLDDT scale. N-terminal region is shown. C) 3D model of crAssE-Sib RBP (shown in tan) superimposed at TaqVP crystal structure pdb 5VF4 (shown in light blue); VR regions of RBD and TaqVP are in green and orange, respectively. Alpha helices (colored in magenta and yellow) show similar orientation of the molecules.
