# Supplementary material for: Genome Analysis of Epsilon CrAss-like Phages
Source: Viruses. 2024 Mar 27;16(4):513. doi: 10.3390/v16040513 (PMC11054128; doi:10.3390/v16040513)
Supplement: Supplementary file 1 [file viruses-16-00513-s001.zip › viruses-2908373-Table S1.pdf]

Table S1: Annotation of the crAssE-Sib genome

#	Putative product	Coordinates, bp	Length, bp	Direction	Type
1.	hypothetical protein	83..289	207	-	CDS
2.	hypothetical protein	312..482	171	-	CDS
3.	hypothetical protein	556..843	288	-	CDS
4.	hypothetical protein	1038..1577	540	-	CDS
5.	hypothetical protein	1938..2105	168	-	CDS
6.	hypothetical protein	2226..2618	393	-	CDS
7.	Phosphodiesterase	2615..3571	957	-	CDS
8.	Polynucleotide kinase, 3 phosphatase	3537..4409	873	-	CDS
9.	hypothetical protein	4409..4819	411	-	CDS
10.	xyloglucanase	5004..5660	657	+	CDS
11.	putative DNA binding protein	5691..5960	270	-	CDS
12.	MmcB-like DNA repair protein	5957..6649	693	-	CDS
13.	tRNA	6667..6738	72	-	tRNA
14.	tRNA	6742..6813	72	-	tRNA
15.	tRNA	6883..6953	71	-	tRNA
16.	tRNA	6955..7027	73	-	tRNA
17.	ATPase	7173..7898	726	-	CDS
18.	dUTPase	7923..8573	651	-	CDS
19.	hypothetical protein	8576..8824	249	-	CDS
20.	ssDNA binding protein	8880..9827	948	-	CDS
21.	DNA polymerase III subunit beta	9840..10,949	1110	-	CDS
22.	Holliday junction resolvase	11,047..11,580	534	-	CDS
23.	hypothetical protein	11,587..12,021	435	-	CDS
24.	hypothetical protein	12,018..12,266	249	-	CDS
25.	hypothetical protein	12,271..12,573	303	-	CDS
26.	Thymidylate synthase	12,570..13,442	873	-	CDS
27.	putative DNA binding protein	13,518..13,718	201	-	CDS
28.	DUF2829 domain-containing protein	13,792..14,061	270	-	CDS
29.	ATP dependent DNA helicase	14,114..15,541	1428	-	CDS
30.	hypothetical protein	15,799..17,367	1569	-	CDS
31.	hypothetical protein	17,421..17,633	213	+	CDS
32.	hypothetical protein	17,717..18,157	441	+	CDS
33.	DNA-binding protein	18,144..18,593	450	+	CDS
34.	HYDIN-like protein	18,609..19,334	726	+	CDS
35.	hypothetical protein	19,338..19,619	282	+	CDS
36.	hypothetical protein	19,606..20,127	522	+	CDS
37.	putative terminase small subunit	20,105..20,443	339	+	CDS
38.	Terminase large subunit	20,406..23,219	2814	+	CDS
39.	Portal protein	23,261..26,020	2760	+	CDS
40.	Structural protein	26,147..27,313	1167	+	CDS
41.	Major capsid protein	27,336..29,036	1701	+	CDS
42.	hypothetical protein	29,112..30,200	1089	+	CDS

43.	Ring protein	30,204..30,878	675	+	CDS
44.	Structural protein	31,021..33,009	1989	+	CDS
45.	hypothetical protein	33,101..34,843	1743	+	CDS
46.	Tetratricopeptide repeat family protein	34,828..35,139	312	+	CDS
47.	Pentaxin family protein	35,139..37,247	2109	+	CDS
48.	hypothetical protein	37,249..37,521	273	+	CDS
49.	Putative structural protein	37,607..37,825	219	+	CDS
50.	Collagen triple helix repeat protein	37,829..38,878	1050	+	CDS
51.	Carbohydrate binding domain protein	38,894..40,006	1113	+	CDS
52.	Peptidase M15	40,137..40,598	462	+	CDS
53.	transmembrain protein	40,600..41,187	588	+	CDS
54.	hypothetical protein	41,184..41,636	453	+	CDS
55.	Structural protein	41,720..42,598	879	+	CDS
56.	hypothetical protein	42,620..42,946	327	+	CDS
57.	hypothetical protein	42,948..43,199	252	+	CDS
58.	Protein of unknown function DUF5977	43,196..43,834	639	+	CDS
59.	receptor binding protein	43,837..45,894	2058	+	CDS
60.	VR	45,751..45,846	96	+	feature
61.	IMH	45,847..45,862	16	+	feature
62.	TR	46,215..46,310	96	+	feature
63.	IMH*	46,311..46,326	16	+	feature
64.	Reverse transcriptase	46,419..47,816	1398	+	CDS
65.	hypothetical protein	47,838..48,230	393	+	CDS
66.	hypothetical protein	48,205..48,483	279	+	CDS
67.	hypothetical protein	48,467..48,892	426	+	CDS
68.	hypothetical protein	48,909..49,295	387	+	CDS
69.	cellulase	49,312..50,091	780	+	CDS
70.	Carbohydrate-binding protein	50,078..54,589	4512	+	CDS
71.	hypothetical protein	54,719..54,841	123	+	CDS
72.	hypothetical protein	54,904..55,320	417	+	CDS
73.	hypothetical protein	55,289..55,678	390	+	CDS
74.	Ring protein	55,675..56,379	705	+	CDS
75.	hypothetical protein	56,451..56,945	495	+	CDS
76.	Phage antirepressor protein	57,049..58,002	954	+	CDS
77.	Ring protein	58,086..58,982	897	+	CDS
78.	RNA-guided endonuclease TnpB family protein	58,986..60,125	1140	-	CDS
79.	hypothetical protein	60,269..60,433	165	+	CDS
80.	Putative tail protein	60,439..61,167	729	+	CDS
81.	hypothetical protein	61,172..62,305	1134	+	CDS
82.	periplasmic metallopeptidase	62,289..62,750	462	+	CDS
83.	hypothetical protein	62,743..63,204	462	+	CDS
84.	hypothetical protein	63,285..64,007	723	+	CDS
85.	hypothetical protein	64,017..64,115	99	+	CDS
86.	RNA-guided endonuclease TnpB family protein	64,190..65,413	1224	+	CDS

87.	Muzzle protein	65,628..72,401	6774	+	CDS
88.	tRNA	72,756..72,828	73	+	tRNA
89.	tRNA	72,901..72,974	74	+	tRNA
90.	Ribonucleoprotein	73,091..74,725	1635	+	CDS
91.	hypothetical protein	74,851..75,264	414	+	CDS
92.	Phage tail lysozyme	75,248..77,668	2421	+	CDS
93.	hypothetical protein	78,420..79,439	1020	+	CDS
94.	Structural protein	79,508..82,348	2841	+	CDS
95.	Structural protein	82,426..85,407	2982	+	CDS
96.	Structural protein	85,400..92,026	6627	+	CDS
97.	hypothetical protein	92,029..92,607	579	+	CDS
98.	DNA-dependent RNA polymerase	92,650..94,908	2259	+	CDS
99.	Repressor protein	95,129..95,353	225	+	CDS
100.	Phage antirepressor protein	95,605..96,489	885	+	CDS
101.	Spike protein/glycoprotein	96,609..97,673	1065	+	CDS
102.	hypothetical protein	97,697..98,110	414	+	CDS
103.	hypothetical protein	98,161..98,655	495	+	CDS
104.	hypothetical protein	98,663..99,040	378	+	CDS
105.	hypothetical protein	99,134..99,427	294	+	CDS
106.	hypothetical protein	99,370..99,549	180	+	CDS
107.	DNA binding protein	99,597..99,767	171	+	CDS
108.	hypothetical protein	99,896..100,123	228	+	CDS
109.	hypothetical protein	100,120..100,335	216	+	CDS
110.	Protein of unknown function (DUF2829)	100,391..101,146	756	+	CDS
111.	hypothetical protein	101,143..101,418	276	+	CDS
112.	hypothetical protein	101,422..101,727	306	+	CDS
113.	hypothetical protein	101,745..102,095	351	+	CDS
114.	hypothetical protein	102,092..102,409	318	+	CDS
115.	hypothetical protein	102,399..102,569	171	+	CDS
116.	hypothetical protein	102,590..102,943	354	+	CDS
117.	hypothetical protein	103,085..103,930	846	+	CDS
118.	Structural protein	103,830..109,487	5658	+	CDS
119.	hypothetical protein	109,702..110,319	618	+	CDS
120.	hypothetical protein	110,324..110,773	450	+	CDS
121.	hypothetical protein	110,888..111,136	249	-	CDS
122.	hypothetical protein	111,129..111,617	489	-	CDS
123.	cell adhesive protein	111,607..111,855	249	-	CDS
124.	hypothetical protein	111,863..112,054	192	-	CDS
125.	hypothetical protein	112,051..112,764	714	-	CDS
126.	hypothetical protein	112,757..113,299	543	-	CDS
127.	DNA (cytosine-5)-methyltransferase	113,296..114,423	1128	-	CDS
128.	hypothetical protein	114,428..114,736	309	-	CDS
129.	dATP/dGTP diphosphohydrolase	114,733..115,290	558	-	CDS
130.	hypothetical protein	115,335..115,676	342	-	CDS
131.	hypothetical protein	115,654..115,959	306	-	CDS
132.	hypothetical protein	115,960..116,316	357	-	CDS
133.	ATP-dependent DNA helicase	116,313..117,734	1422	-	CDS

134.	hypothetical protein	117,724..118,110	387	-	CDS
135.	hypothetical protein	118,117..118,440	324	-	CDS
136.	hypothetical protein	118,440..118,610	171	-	CDS
137.	exodeoxyribonuclease	118,607..119,626	1020	-	CDS
138.	hypothetical protein	119,623..119,982	360	-	CDS
139.	hypothetical protein	120,007..120,159	153	-	CDS
140.	flotillin family protein	120,146..121,846	1701	-	CDS
141.	hypothetical protein	121,879..122,373	495	-	CDS
142.	acyl carrier protein	122,363..122,614	252	-	CDS
143.	hypothetical protein	122,602..123,315	714	-	CDS
144.	hypothetical protein	123,318..123,515	198	-	CDS
145.	hypothetical protein	123,531..124,286	756	-	CDS
146.	DNAB-Like Replicative Helicase; ATPase	124,148..125,272	1125	-	CDS
147.	hypothetical protein	125,250..125,528	279	-	CDS
148.	zinc-finger-containing domain protein	125,580..126,146	567	-	CDS
149.	hypothetical protein	126,113..126,370	258	-	CDS
150.	hypothetical protein	126,375..126,728	354	-	CDS
151.	DNA primase/helicase	126,706..127,719	1014	-	CDS
152.	DNA replication factor	127,742..129,058	1317	-	CDS
153.	hypothetical protein	129,055..129,231	177	-	CDS
154.	NinG protein	129,215..129,685	471	-	CDS
155.	hypothetical protein	129,685..129,936	252	-	CDS
156.	DUF4494 domain-contain protein	129,929..130,399	471	-	CDS
157.	hypothetical protein	130,411..130,671	261	-	CDS
158.	hypothetical protein	130,740..131,408	669	-	CDS
159.	hypothetical protein	131,405..131,608	204	-	CDS
160.	P63C domain-containing protein	131,605..131,793	189	-	CDS
161.	hypothetical protein	131,801..131,953	153	-	CDS
162.	hypothetical protein	131,999..132,265	267	-	CDS
163.	hypothetical protein	132,310..132,480	171	-	CDS
164.	hypothetical protein	132,564..132,815	252	-	CDS
165.	hypothetical protein	132,833..133,129	297	-	CDS
166.	hypothetical protein	133,303..133,614	312	-	CDS
167.	hypothetical protein	133,635..133,886	252	-	CDS
168.	hypothetical protein	133,911..134,294	384	-	CDS
169.	hypothetical protein	134,297..134,605	309	-	CDS
170.	AAA ATPase domain protein	134,617..135,414	798	-	CDS
171.	hypothetical protein	135,411..135,632	222	-	CDS
172.	DUF3988 domain-containing protein	135,757..136,593	837	-	CDS
173.	hypothetical protein	136,605..137,042	438	-	CDS
174.	Poly A polymerase	137,063..137,725	663	-	CDS
175.	hypothetical protein	137,744..137,998	255	-	CDS
176.	hypothetical protein	138,006..138,482	477	-	CDS
177.	hypothetical protein	138,482..138,706	225	-	CDS
178.	hypothetical protein	138,777..139,958	1182	-	CDS
179.	hypothetical protein	139,963..140,703	741	-	CDS

180.	hypothetical protein	140,744..141,805	1062	-	CDS
181.	hypothetical protein	141,818..142,108	291	-	CDS
182.	hypothetical protein	142,117..143,013	897	-	CDS
183.	hypothetical protein	143,025..143,279	255	-	CDS
184.	hypothetical protein	143,270..143,575	306	-	CDS
185.	hypothetical protein	143,577..143,969	393	-	CDS
186.	hypothetical protein	144,007..144,357	351	-	CDS
187.	hypothetical protein	144,354..144,605	252	-	CDS
188.	hypothetical protein	144,685..145,083	399	-	CDS
189.	Lys_methyl_FliB super family protein	145,073..145,546	474	-	CDS
190.	hypothetical protein	145,543..145,815	273	-	CDS
191.	hypothetical protein	145,812..146,195	384	-	CDS
192.	hypothetical protein	146,146..146,439	294	-	CDS
193.	hypothetical protein	146,420..146,704	285	-	CDS
194.	hypothetical protein	146,732..147,043	312	-	CDS
195.	hypothetical protein	147,063..147,536	474	-	CDS
196.	hypothetical protein	147,533..147,787	255	-	CDS
197.	hypothetical protein	147,907..148,140	234	-	CDS
198.	hypothetical protein	148,121..148,546	426	-	CDS
199.	hypothetical protein	148,558..148,716	159	-	CDS
200.	hypothetical protein	148,722..149,036	315	-	CDS
201.	hypothetical protein	149,045..149,392	348	-	CDS
202.	hypothetical protein	149,389..149,793	405	-	CDS
203.	hypothetical protein	149,942..150,061	120	-	CDS
204.	hypothetical protein	150,130..150,390	261	-	CDS
205.	hypothetical protein	150,547..150,669	123	-	CDS
206.	hypothetical protein	150,800..150,985	186	-	CDS
