# Supplementary material for: Genome Analysis of Epsilon CrAss-like Phages
Source: Viruses. 2024 Mar 27;16(4):513. doi: 10.3390/v16040513 (PMC11054128; doi:10.3390/v16040513)
Supplement: Supplementary file 1 [file viruses-16-00513-s001.zip › viruses-2908373-Table S2.pdf]

Table S2: List of the epsilon crAss-like phages noted in Figures 2, 3, 4

#	GenBank accession number	Isolate	Length, bp
1.	OR575929	crAssE-Sib	151256
2.	OP074661	2523_57290	152120
3.	OP072923	PF-P004_88583	146609
4.	OP072905	4298_68366	150730
5.	BK053219	ctCP22	155962
6.	OP074781	2657_49232	145370
7.	OP075230	3126_50468	149939
8.	OP076321	1914_29733	148608
9.	OP030796	0498_72044	150546
10.	OP075812	3801_30080	152036
11.	OP075003	2901_48968	156437
12.	OP030781	0359_54031	147673
13.	OP073967	1482_59065	157702
14.	OP075874	3870_22255	151190
15.	OP075824	3814_49619	156965
16.	OP072453	2917_22467	144605
17.	OP075288	3192_57230	151983
18.	OP076183	4254_70450	147544
19.	OP072437	2884_41581	147208
20.	OP074405	2207_27220	144250
21.	BK053219	ctCP22	155962
22.	BK023142	ctVE36	158714
23.	BK031280	ctfN46	144113
24.	BK027871	ctqVY12	144367
25.	BK038654	ct8FW29	148799
26.	BK048677	ctH0215	151346
27.	BK052849	ctvdV13	158723
28.	BK037909	ctslE2	156641
29.	BK057374	ctR3g24	151391
30.	OFPS01000054	OFPS01000054	144391
31.	OICR01000007	OICR01000007	146329
32.	OMGY01000027	OMGY01000027	147101
33.	OFLY01000012	OFLY01000012	147368
34.	OLJW01000056	OLJW01000056	148853
35.	OJUG01000072	OJUG01000072	148883
36.	OJSE01000052	OJSE01000052	148953
37.	OHXK01000005	OHXK01000005	149835
38.	OGZF01000025	OGZF01000025	150604
39.	OHBE01000017	OHBE01000017	150646
40.	OMDY01000016	OMDY01000016	151112
41.	OLZO01000021	OLZO01000021	151133
42.	OIWQ01000029	OIWQ01000029	151719
43.	OHMY01000035	OHMY01000035	151909
44.	OHKV01000027	OHKV01000027	151910
45.	OHQS01000030	OHQS01000030	151910
46.	OCHF01000011	OCHF01000011	151921
47.	OCPY01000015	OCPY01000015	151921
48.	OCPZ01000017	OCPZ01000017	151921

49.	OGOO01000044	OGOO01000044	151997
50.	OIZE01000030	OIZE01000030	152163
51.	OHEN01000023	OHEN01000023	152275
52.	OIAT01000008	OIAT01000008	152556
53.	OFMT01000022	OFMT01000022	153190
54.	OJOU01000011	OJOU01000011	153255
55.	OFMU01000028	OFMU01000028	153731
56.	OBWI01000023	OBWI01000023	153977
57.	PPYF01266999	PPYF01266999	154058
58.	OICG01000006	OICG01000006	154154
59.	OLOZ01000027	OLOZ01000027	154420
60.	OGZB01000001	OGZB01000001	154871
61.	OLOG01000029	OLOG01000029	155271
62.	OLQV01000028	OLQV01000028	156426
63.	OGZF01000022	OGZF01000022	158368
64.	OHYE01000011	OHYE01000011	158763
65.	OJQQ01000027	OJQQ01000027	158910
